# Toxicity Index, patient-reported outcomes, and persistence of breast cancer chemotherapy-associated side effects in NRG Oncology/NSABP B-30

**DOI:** 10.1038/s41523-022-00489-9

**Published:** 2022-11-19

**Authors:** N. Lynn Henry, Sungjin Kim, Ron D. Hays, Marcio A. Diniz, Mourad Tighiouart, Gillian Gresham, Michael Luu, Reena S. Cecchini, Greg Yothers, André Rogatko, Patricia A. Ganz

**Affiliations:** 1 Department of Internal Medicine, University of Michigan Medical School, Ann Arbor, MI USA; 2 Biostatistics and Bioinformatics Research Center, Cedars-Sinai Cancer Center, Los Angeles, CA USA; 3 Department of Medicine, David Geffen School of Medicine, University of California Los Angeles, Los Angeles, CA USA; 4 Department of Health Policy & Management, Fielding School of Public Health, University of California Los Angeles, Los Angeles, CA USA; 5 Department of Medicine, Cedars-Sinai Cancer Center, Los Angeles, USA; 6 Department of Biostatistics, University of Pittsburgh, Pittsburgh, PA USA; 7 Jonsson Comprehensive Cancer Center, University of California Los Angeles, Los Angeles, CA USA

**Keywords:** Signs and symptoms, Breast cancer, Chemotherapy

## Abstract

Adjuvant chemotherapy improves breast cancer survival but is associated with bothersome short- and long-term toxicity. Factors associated with toxicity, especially subacute toxicity up to 2 years following chemotherapy, have not been fully elucidated. The NRG Oncology/NSABP B-30 clinical trial compared 3 different doxorubicin-, cyclophosphamide-, and docetaxel-based chemotherapy regimens given over 3–6 months. Patients with hormone receptor-positive breast cancer received subsequent adjuvant endocrine therapy. From baseline through 24 months, 2156 patients completed questionnaires serially. We used multivariable probabilistic index models to identify factors associated with acute (>0–12 months) and subacute (>12–24 months) difficulties with pain, cognition, vasomotor symptoms, and vaginal symptoms. For all symptom domains, presence of symptoms prior to chemotherapy initiation were associated with symptoms in the subacute period (all *p* < 0.001). In addition, different combinations of patient factors and breast cancer treatments were associated with increased likelihood of pain, vasomotor, and vaginal symptoms in the subacute period. Consideration of pre-treatment symptoms and patient factors, as well as treatments for breast cancer, can facilitate identification of groups of patients that may experience symptoms following completion of chemotherapy. This information may be important for treatment-decision-making when alternative regimens are equivalent in benefit.

## Introduction

The introduction of taxane-based chemotherapy has resulted in improvements in disease-free and overall survival in breast cancer^1^. However, these treatments can cause long-lasting symptoms that have detrimental effects on health-related quality of life^2,3^. Identifying patient-level variables associated with treatment tolerability is important for treatment-decision-making.

Between 1999 and 2004, 5351 patients with breast cancer at high risk of recurrence enrolled in the NRG Oncology/NSABP B-30 clinical trial and were randomized to one of three adjuvant chemotherapy regimens: (1) doxorubicin (A), cyclophosphamide (C), and docetaxel (T) given concurrently (ATC), (2) sequential doxorubicin and cyclophosphamide followed by docetaxel (AC->T), or (3) doxorubicin and docetaxel (AT)^4^. Patients with hormone receptor-positive breast cancer were treated with adjuvant endocrine therapy, 79% with tamoxifen and 21% with anastrozole. Disease-free survival was 17–20% better for patients treated with the sequential AC->T regimen compared to the other two regimens, and overall survival was improved by 17% for AC->T compared to AT. However, both bother from symptoms (total symptom score) and overall health-related quality of life (HRQOL) 6 months after baseline were statistically significantly worse for the AC-T group than for the other two groups^5^. Patients in all three treatment groups continued to report increased bother from symptoms up to 2 years after chemotherapy was completed compared to baseline, although overall HRQOL had returned to baseline at 12 months^5^.

We have developed novel methods to summarize and analyze toxicity, allowing for a more complete account of a patient’s symptom experience^6–9^. When applied to patient-reported outcomes data, the Toxicity Index (TI) considers the severity of bother from symptoms to generate a summary score that reflects the impact of multiple symptoms experienced by an individual patient over time.

Identifying characteristics of patients at greater risk of symptoms following treatment who may benefit from more intensive monitoring and management of toxicity may lead to improved long-term HRQOL for cancer survivors. Therefore, as part of the US National Cancer Institute Cancer Moonshot research initiative, we conducted this secondary analysis from the NSABP B-30 clinical trial to explore factors associated with symptoms in the 1–2 years following adjuvant chemotherapy.

## Results

### Study population

**Fig. 1:**
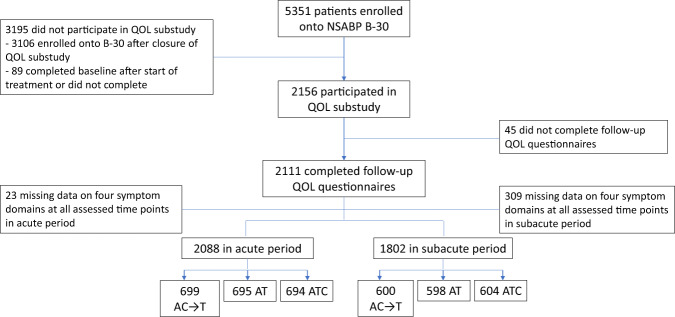
CONSORT diagram. A doxorubicin, C cyclophosphamide, QOL quality of life, T docetaxel.

A total of 2156 patients were enrolled in the QOL substudy, evenly divided across the three treatment regimens (Fig. 1). Baseline characteristics of the analyzed patients (2088 in acute and 1802 in subacute period) are presented in Table 1. Fewer patients completed the symptom checklist during the second year following registration, although more than 70% of patients in each treatment arm had patient-reported symptom data at each timepoint (Supplementary Information Table 1). There were no differences in baseline characteristics between patients with and without patient-reported data during the second year, other than more patients who received adjuvant hormonal therapy completed the questionnaires (Supplementary Information Table 2).

**Table 1:** Baseline patient characteristics stratified by treatment group and period.

Variable	Acute period				Subacute period			
	All (*N* = 2088)	AC- >T (*N* = 699)	AT (*N* = 695)	ATC (*N* = 694)	All (*N* = 1802)	AC- >T (*N* = 600)	AT (*N* = 598)	ATC (*N* = 604)
Age at surgery (years), median (IQR)	49 (43–57)	50 (43–57)	49 (43–56)	50 (44–57)	50 (44–57)	49.5 (43–57)	49 (43–56)	51 (45–58)
Age at surgery (years)								
[24, 43]	457 (21.9%)	157 (22.5%)	157 (22.6%)	143 (20.6%)	384 (21.3%)	140 (23.3%)	134 (22.4%)	110 (18.2%)
[43, 50]	589 (28.2%)	190 (27.2%)	211 (30.4%)	188 (27.1%)	504 (28%)	160 (26.7%)	177 (29.6%)	167 (27.7%)
[50, 57]	501 (24%)	155 (22.2%)	171 (24.6%)	175 (25.2%)	441 (24.5%)	136 (22.7%)	152 (25.4%)	153 (25.3%)
[57, 80]	541 (25.9%)	197 (28.2%)	156 (22.5%)	188 (27.1%)	473 (26.3%)	164 (27.3%)	135 (22.6%)	174 (28.8%)
Race/ethnicity								
NH White	1796 (86.0%)	593 (84.8%)	597 (85.9%)	606 (87.3%)	1564 (86.8%)	520 (86.7%)	512 (85.7%)	532 (88.1%)
Hispanic	59 (2.8%)	19 (2.7%)	20 (2.9%)	20 (2.9%)	44 (2.4%)	10 (1.7%)	16 (2.7%)	18 (3%)
NH Black	172 (8.2%)	65 (9.3%)	53 (7.6%)	54 (7.8%)	142 (7.9%)	53 (8.8%)	46 (7.7%)	43 (7.1%)
Other	60 (2.9%)	22 (3.2%)	24 (3.5%)	14 (2%)	51 (2.8%)	17 (2.8%)	23 (3.9%)	11 (1.8%)
Unknown	1 (0.05%)	0 (0)	1 (0.1%)	0 (0)	1 (0.06%)	0 (0)	1 (0.2%)	0 (0)
Body mass index (kg/m^2^)								
Underweight BMI < 18.5	24 (1.2%)	8 (1.1%)	6 (0.9%)	10 (1.4%)	20 (1.1%)	7 (1.2%)	5 (0.8%)	8 (1.3%)
Normal weight 18.5 ≤ BMI < 25	764 (36.6%)	260 (37.2%)	258 (37.1%)	246 (35.5%)	663 (36.8%)	224 (37.3%)	219 (36.6%)	220 (36.4%)
Overweight 25 ≤ BMI < 30	675 (32.3%)	217 (31%)	222 (31.9%)	236 (34%)	578 (32.1%)	191 (31.8%)	188 (31.4%)	199 (33%)
Obesity BMI ≥ 30	625 (29.9%)	214 (30.6%)	209 (30.1%)	202 (29.1%)	541 (30%)	178 (29.7%)	186 (31.1%)	177 (29.3%)
Type of surgery								
Lumpectomy	1071 (51.3%)	362 (51.8%)	349 (50.2%)	360 (51.9%)	922 (51.2%)	303 (50.5%)	307 (51.3%)	312 (51.7%)
Mastectomy	1017 (48.7%)	337 (48.2%)	346 (49.8%)	334 (48.1%)	880 (48.8%)	297 (49.5%)	291 (48.7%)	292 (48.3%)
Hormonal therapy								
Yes	1654 (79.2%)	553 (79.1%)	553 (79.6%)	548 (79%)	1455 (80.7%)	484 (80.7%)	486 (81.3%)	485 (80.3%)
No	434 (20.8%)	146 (20.9%)	142 (20.4%)	146 (21%)	347 (19.3%)	116 (19.3%)	112 (18.7%)	119 (19.7%)

Data are presented as number of patients (column %) or median (*IQR* interquartile range).

*A* doxorubicin, *BMI* body mass index, *C* cyclophosphamide, *NH* Non-Hispanic, *T* docetaxel.

### Symptom domains and individual symptoms

We examined factors associated with increased risk of experiencing acute and subacute problems for each symptom domain and for individual symptoms.

#### Pain domain

**Fig. 2:**
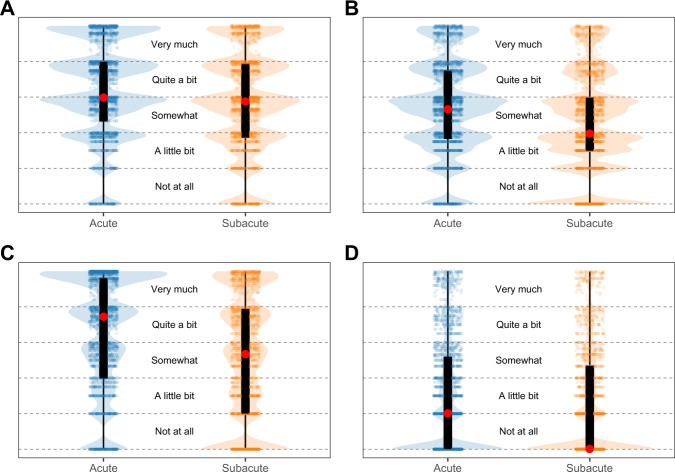
Violin plots demonstrating the distribution of Toxicity Index in the acute (blue) and subacute (orange) time periods for each symptom domain. Red circles represent the median, and black bars represent the interquartile range. **A** pain. **B** cognition. **C** vasomotor. **D** vaginal.

The pain domain included the patient-reported symptoms of numbness, general aches and pains, joint pains, swelling of hands or feet, and muscle stiffness. As demonstrated in the violin plots in Fig. 2A, the median pain level designated by the red circle was “quite a bit” in both the acute and subacute time periods; the black rectangle depicts the interquartile range (IQR). The width of the blue or orange bars reflects the number of patients with each TI value on the *y*-axis. Therefore, when comparing the acute and subacute periods for the pain domain, although the median values and IQR are similar between the two periods, there is a shift toward lower TI values in the subacute period, as can be seen in the increased width of the orange bars compared to the blue bars toward the bottom of the figure.

**Fig. 3:**
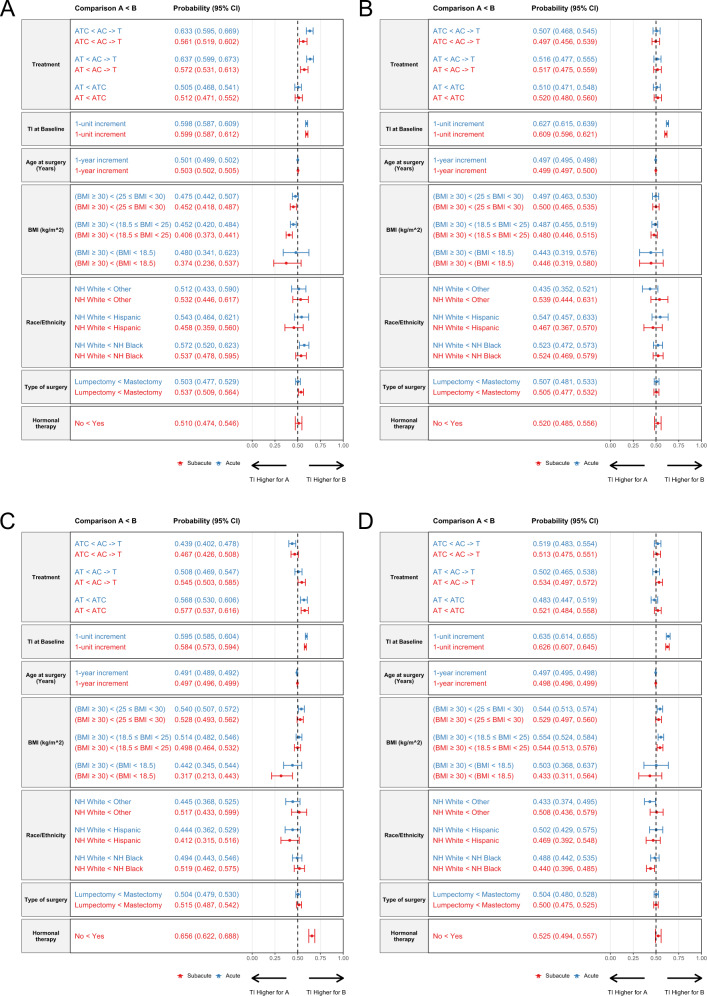
Forest plots demonstrating results of Multivariable Probabilistic Index Models for the cumulative Toxicity Index (TI) for the symptom domains during the acute and subacute time periods. Comparison A < B denotes the probability that the cumulative TI for B is higher than that for A. When the probability is >0.5, B is more likely to be associated with the symptom domain, whereas when the probability is <0.5, A is more likely to be associated with the symptom domain. **A** pain domain. Acute (blue, *N* = 2075 patients) and subacute (red, *N* = 1791) time periods. **B** cognition domain. Acute (blue, *N* = 2078 patients) and subacute (red, *N* = 1793) time periods. **C** vasomotor domain. Acute (blue, *N* = 2075 patients) and subacute (red, *N* = 1791) time periods. **D** vaginal domain. Acute (blue, *N* = 2068 patients) and subacute (red, *N* = 1781) time periods. A doxorubicin, BMI body mass index, C cyclophosphamide, CI confidence interval, NH Non-Hispanic, T docetaxel, TI toxicity index.

The multivariable probabilistic index models (PIM) for the cumulative TI for the pain domain included each of the factors listed in Fig. 3A and Supplementary Information Table 3. For each comparison, probability values closer to 0.5 reflect less difference between the compared factors. However, when values are substantially less than 0.5, the first factor listed (“A”) is more likely to be associated with increased risk of the symptom of interest, whereas when values are greater than 0.5, the second factor listed (“B”) is more likely to be associated with increased risk of the symptom of interest.

For example, as shown in Fig. 3A, during the acute period there was no difference in pain between the AT and ATC chemotherapy regimens, with a probability of 0.505 (95% CI 0.468–0.541, *P* = 0.768). However, differences were noted for both the AT vs AC->T comparison and the ATC vs AC->T comparison (probability 0.637 [95% CI 0.599–0.673], *P* < 0.001 and 0.633 [95% CI 0.595–0.669], *P* < 0.001, respectively). In both cases, the probabilities are greater than 0.5 and therefore AC->T (the “B” factor) was associated with increased risk of pain in the acute time periods. In addition, pain at baseline (*P* < 0.001), obesity (compared to normal weight, *P* = 0.004), and Black race (*p* = 0.007) were all associated with increased likelihood of pain during the acute period.

In contrast, during the subacute period, treatment with the sequential AC->T chemotherapy regimen (*P* = 0.001), pain at baseline (*P* < 0.001), obesity (compared to normal, *P* < 0.001; compared to overweight, *P* = 0.007), older age at surgery (*P* < 0.001), and mastectomy (*P* = 0.009), but not Black race (*P* = 0.224), were associated with increased likelihood of pain.

Each individual item in the pain domain was also examined separately (Supplementary Information Tables 4 and 5). Similar to the pain domain model, treatment with a cyclophosphamide-containing chemotherapy regimen (*P*-values 0.024–<0.001) and presence of the symptom at baseline (all *P* < 0.001) were both associated with increased likelihood of each of the individual pain symptoms during the acute period. In addition, obesity was associated with an increased likelihood of numbness (*P* = 0.007), general aches and pains (*P* = 0.006), and swelling of the hands and feet (*P* < 0.001), and Black race was associated with an increased likelihood of numbness (*P* < 0.001). Age and breast surgery type were not associated with increased likelihood of any of the five individual symptoms.

During the subacute period, however, only presence of each individual symptom at baseline and obesity were associated with likelihood of each of the five items in the pain domain. AC->T relative to AT remained associated with an increased likelihood of numbness (*P* < 0.001), joint pain (*P* = 0.003), and muscle stiffness (*P* = 0.044). Patients in the oldest quartile of age had a higher likelihood of all symptoms other than swelling of the hands and feet relative to those in the youngest quartile (*P*-values 0.003–<0.001). Mastectomy, as opposed to lumpectomy, was associated with increased likelihood of numbness (*P* < 0.001), joint pain (*P* = 0.018), and swelling of the hands and feet (*P* = 0.039). Use of hormonal therapy was only associated with increased likelihood of swelling of the hands and feet (*P* = 0.043).

#### Cognition domain

The cognition domain included patient-reported symptoms of mood swings, forgetfulness, and difficulty concentrating. As demonstrated in the violin plots, patients reported cognitive symptoms in the “somewhat” range during the acute period, which decreased to the “a little bit” range in the subacute time period, and the IQR shifted downward (Fig. 2B). In addition, there were fewer patients reporting TI values in the “very much,” “quite a bit,” and “somewhat” ranges in the subacute period compared to the acute period. Only cognitive symptoms at baseline (*P* < 0.001) and younger age at surgery (*P* < 0.001) were associated with increased likelihood of cognitive problems during the acute period (Fig. 3B, Supplementary Information Table 6). During the subacute period, only cognitive symptoms at baseline were associated with increased likelihood of cognitive symptoms (*P* < 0.001).

Each individual item in the cognition domain was also examined separately (Supplementary Information Tables 7 and 8). As seen in the overall cognition domain model, presence of the symptom at baseline (*P* < 0.001) and younger age were both associated with increased likelihood of individual cognitive symptoms during the acute period. In particular, patients in the two youngest age quartiles (age 24–50) were at increased risk for both mood swings (*P* < 0.001) and forgetfulness (*P* < 0.005, *P* < 0.015) compared to those in the oldest age quartile (age 57–80). In contrast, patients in the middle two age quartiles (age 43–57) were at increased risk for difficulty concentrating (*P* = 0.010, *P* = 0.013). Black race (*P* = 0.038) was associated with an increased risk of mood swings during the acute period.

In the subacute period, presence of the symptom at baseline (*P* < 0.001) was associated with increased likelihood of all three individual cognitive symptoms. Younger age (age 24–50 vs age 57–80, *P* < 0.001 and *P* = 0.015) was only associated with increased likelihood of mood swings. No other associations were identified, including with race or with use of hormone therapy.

#### Vasomotor domain

The vasomotor domain included patient-reported symptoms of hot flashes, night sweats and cold sweats. As demonstrated in the violin plots, patients reported vasomotor symptoms in the “quite a bit” range during the acute period, which decreased to the “somewhat” range in the subacute time period, and the IQR shifted downward (Fig. 2C). In addition, there were fewer patients reporting TI values in the “very much” and “quite a bit” ranges in the subacute period compared to the acute period.

Treatment with the ATC chemotherapy regimen (*P* < 0.001), baseline vasomotor symptoms (*P* < 0.001), younger age (*P* < 0.001), and overweight (compared to obesity, *P* < 0.018) were all associated with increased likelihood of vasomotor symptoms during the acute period (Fig. 3C, Supplementary Information Table 9). Similarly, during the subacute period, treatment with a cyclophosphamide-containing chemotherapy regimen (ATC vs AT, *P* < 0.001; AC->T vs AT, *P* = 0.019), baseline vasomotor symptoms (*P* < 0.001), younger age (*P* = 0.001), and obesity (compared to underweight, *P* = 0.005), as well as receipt of hormonal therapy (*P* < 0.001), were associated with increased likelihood of vasomotor symptoms.

For hot flashes and night sweats individually, similar to the vasomotor domain model, treatment with the concurrent ATC chemotherapy regimen (*P* < 0.001–*P* = 0.004), baseline symptoms (*P* < 0.001), younger age (*P* < 0.001), and overweight (compared to obesity, *P* = 0.015–0.026) were all associated with increased likelihood of hot flashes during the acute period (Supplementary Information Table 10). In addition, white race (compared to other, *P* = 0.019) was associated with increased likelihood of night sweats.

During the subacute period, treatment with ATC chemotherapy regimen (vs AT, *P* = 0.001), baseline symptoms (*P* < 0.001), younger age, and receipt of hormonal therapy (*P* < 0.001), but not overweight status (*P* = 0.056), were associated with increased likelihood of hot flashes (Supplementary Information Table 11). When examining the age associations and hot flashes, only the middle two age quartiles (age 43–57, *P* < 0.001) but not the youngest patients (*P* = 0.48) were at increased risk relative to the oldest age quartile (age 57–80). Similarly, treatment with ATC chemotherapy regimen (vs AT, *p* = 0.001), baseline symptoms (*P* < 0.001), younger age (*P* < 0.001–*P* = 0.034), obesity (vs underweight, *P* < 0.002), and receipt of hormonal therapy (*P* < 0.001) were associated with increased likelihood of night sweats.

#### Vaginal domain

The vaginal domain consisted of pain with intercourse and vaginal dryness. As demonstrated in the violin plots, patients reported vaginal symptoms in the “a little bit” range during the acute period, which decreased to the “not at all” range in the subacute time period, and the IQR shifted downward (Fig. 2D).

Baseline vaginal symptoms (*P* < 0.001), younger age (*P* < 0.001), white race (vs other, *P* = 0.033), normal weight (vs obese, *P* < 0.001), and overweight (vs obese, *P* = 0.005) were all associated with increased likelihood of vasomotor symptoms during the acute period (Fig. 3D, Supplementary Information Table 12). Similarly, during the subacute period, baseline vaginal symptoms (*P* < 0.001), younger age (*P* < 0.001), white race (vs Black, *P* = 0.009), and normal weight (vs obese, *P* = 0.006), but not receipt of hormonal therapy (*P* = 0.12), were associated with increased likelihood of vaginal symptoms.

For vaginal dryness and pain with intercourse individually, similar to the vaginal symptom domain model, baseline symptoms (*P* < 0.001), younger age (*P* < 0.001–*P* = 0.008), and non-obese weight (*P* < 0.001–*P* = 0.012) were all associated with increased likelihood of vaginal dryness and pain with intercourse during the acute period (Supplementary Information Table 13). In addition, white race (vs other, *P* = 0.002) was also associated with increased likelihood of vaginal dryness. Similarly, during the subacute period, baseline vaginal dryness (*P* < 0.001), white race (vs Black, *P* = 0.019), and normal weight (vs obese, *P* = 0.004), but not receipt of hormonal therapy (*P* = 0.343), were associated with increased likelihood of vaginal dryness (Supplementary Information Table 14). When examining the age associations and vaginal dryness, only the middle two age quartiles (age 43–57, *P* = 0.002 and *P* = 0.029) but not the youngest patients were at increased risk relative to the oldest age quartile (age 57–80). Similarly, baseline pain with intercourse (*P* < 0.001), white race (vs Black, *P* < 0.001), younger age (*P* < 0.001–*P* = 0.042), and normal weight (vs obese, *P* = 0.001), as well as receipt of hormonal therapy (*P* = 0.004) and AC-T (vs AT, *P* = 0.008), were associated with increased likelihood of pain with intercourse.

## Discussion

This study assessed symptoms in the acute and subacute time frames after treatment, as well as patient-level variables associated with tolerability of adjuvant chemotherapy. By including presence of symptoms at the time of chemotherapy initiation in the models, we were able to investigate whether pre-existing symptoms are associated with treatment toxicity, and which patients are likely to become or remain more symptomatic. In addition, we identified different patterns of symptoms in the acute versus subacute time periods, as demonstrated in the violin plots (Fig. 2). These findings extend previous reports from the NSABP B-30 clinical trial that demonstrated that patients continued to report symptoms in the year after treatment with adjuvant chemotherapy^5^. For neuropathy in particular, our findings are consistent with a prior analysis of B-30 clinical trial data that was performed using a different methodology^10^. In addition, presence of individual symptoms following completion of adjuvant chemotherapy had not previously been evaluated.

Our findings demonstrate different associations between patient-level variables and presence of symptoms in the acute and subacute periods across different symptom domains. For example, multiple factors were associated with pain. Baseline pain is significantly associated with pain following treatment. In addition, if a patient is older, obese, and undergoes a mastectomy, she may have a more difficult time recovering from treatment and may be more likely to have numbness as well as joint pain in the subacute period. In contrast, for the three cognitive symptoms, only baseline symptoms and, for mood swings, younger age, were associated with symptoms in the subacute period.

Some of the findings were expected. For example, during both the acute and subacute periods, vaginal dryness and pain with intercourse were both more commonly reported by patients who were not obese, likely because of lower levels of circulating estrogen in non-obese compared to obese postmenopausal women^11^. We also found that women age 57 and younger at diagnosis had a higher likelihood of vaginal dryness in the acute period compared with older women, presumably because of chemotherapy-induced ovarian suppression in the younger women who were still pre- or perimenopausal at diagnosis. However, in the subacute period the women in the youngest quartile no longer had a higher likelihood of vaginal dryness compared to the oldest quartile, likely because of higher rates of ovarian function recovery following chemotherapy in women age 40 and younger^5^. Younger women in particular continued to have a higher likelihood of pain with intercourse in the subacute period, although it is unknown whether this finding may be related to differences in sexual activity among age groups. Tamoxifen use was also associated with higher likelihood of pain with intercourse, but not with vaginal dryness.

Importantly, the vast majority of patients treated with endocrine therapy in this clinical trial received tamoxifen, since it was the standard of care at the time of enrollment for the majority of patients. Therefore, this choice of endocrine therapy likely influenced the findings related to the pain, vaginal, and vasomotor domains. Use of endocrine therapy was not associated with either vaginal dryness or joint pain, but was associated with hot flashes and night sweats. Consistent with the results from the NSABP P-1 clinical trial of tamoxifen versus placebo, there was also no association between use of endocrine therapy and mood swings^12^.

Strengths of this analysis include patient-reported outcomes collected from participants in a large, randomized clinical trial of women starting treatment with adjuvant chemotherapy for breast cancer. In addition, there is an advantage of using the TI for this analysis because of the cumulative assessment of toxicity over time. There are some limitations to our analyses using the TI method, since patients with missing data in the second year of the study had to be excluded from the analysis of the subacute period. However, even in month 24 more than 73% of patients in all study arms provided patient-reported symptom data, so this limitation was minimized. In addition, information about adherence to adjuvant endocrine therapy, ovarian function following chemotherapy, and concomitant medications was either not collected or was incomplete; all of these factors can influence a patient’s symptoms.

In summary, evaluation of pre-treatment symptoms, multiple patient factors, and use of other treatment modalities can permit identification of which patients are likely to tolerate chemotherapy, and which ones may experience symptoms following completion of treatment. This report adds to a growing body of literature that has identified the importance of considering pre-treatment symptoms and patient risk factors for post-treatment symptoms such as fatigue, as well as tolerance of long-term adjuvant therapies^13–16^. This information may be important for clinicians and patients as they make decisions about chemotherapy when several alternative regimens are equivalent in benefit.

## Methods

### Patients

We used patient-reported data from those enrolled in the “QOL substudy” of the NRG Oncology/NSABP B-30 clinical trial (NCT00003782). Eligible patients had stage 2 or 3 breast adenocarcinoma and had undergone primary surgery with lumpectomy or mastectomy and axillary lymph node dissection. Ineligibility criteria included prior treatment with anthracycline- or taxane-chemotherapy, previous treatment for breast cancer, or grade 2 or higher peripheral neuropathy. The protocol was Institutional Review Board-approved at all participating sites, and all patients provided written informed consent. Patients were randomized 1:1:1 to one of three intravenous chemotherapy regimens: sequential A 60 mg/m^2^ plus C 600 mg/m^2^ every 3 weeks for four cycles followed by T 100 mg/m^2^ every 3 weeks for four cycles (AC->T); concurrent A 60 mg/m^2^, C 600 mg/m^2^, plus T 60 mg/m^2^ every 3 weeks for four cycles (ATC); and A 60 mg/m^2^ plus T 60 mg/m^2^ every 4 weeks for four cycles (AT). For patients with hormone receptor-positive tumors, tamoxifen 20 mg orally daily was started concurrently with chemotherapy. Of note, in September 2000, for safety reasons the ATC regimen was modified to A 50 mg/m^2^, C 500 mg/m^2^, plus T 75 mg/m^2^ every 3 weeks for four cycles and the AT regimen was modified to A 50 mg/m^2^ plus T 75 mg/m^2^ every 4 weeks for four cycles, both with primary growth factor prophylaxis. In October 2002, tamoxifen initiation was delayed until after completion of chemotherapy, and postmenopausal women had the option of taking anastrozole 1 mg orally daily instead of tamoxifen.

### Procedures

Per protocol, the first 2100 consecutively enrolled patients were included in the QOL substudy and asked to complete questionnaires to assess symptoms before chemotherapy, at cycle 4 day 1 of chemotherapy, at 6 months, and then every 6 months through 24 months following chemotherapy initiation. Twenty-four symptoms from the Breast Cancer Prevention Trial symptom checklist^17^, in combination with 4 additional symptoms applicable to chemotherapy and endocrine therapy (mouth sores, skin problems, fever/shivering, and mood swings) were queried. Five response options were offered to report bother from each symptom, ranging from “Not at All” to “Very Much” over the past 7 days.

### Study design and analysis

The acute period was defined as cycle 4 day 1 of chemotherapy through the 12-month follow-up visit and included data collected at cycle 4 day 1, the 6-month visit, and the 12-month visit (Supplementary Information Fig. 1). The subacute period was defined as the time from 12 to 24 months after randomization, including data collected at the 18- and 24-month follow-up visits.

A scree plot of eigenvalues and parallel analyses^18^ suggested four underlying symptom domains from the symptom checklist items: (1) 5-item pain domain (joint pains; general aches and pains; muscle stiffness; swelling of hands and feet; numbness or tingling in hands or feet); (2) 3-item cognition domain (difficulty concentrating; forgetfulness; mood swings); (3) 3-item vasomotor domain (night sweats; hot flashes; cold sweats); and (4) 2-item vaginal domain (vaginal dryness; pain with intercourse). These domains largely replicate other analyses done with this symptom checklist^17,19^.

The cumulative TI is a summary score that assesses the impact of multiple toxicities experienced by an individual patient over a period time by accounting for the frequency and severity of symptoms during that time^6,8,9^. Individual responses reported during the acute period and the subacute period were separately summarized using the cumulative TI. The cumulative TI was considered missing if a patient’s response was missing at all assessed time points in the given period.

Analyses of the cumulative TI at the domain-level (pain, cognition, vasomotor, vaginal) and individual symptom-level were conducted for each period using probabilistic index models (PIM)^20–24^ to identify factors associated with increased risk of experiencing symptoms. Models were adjusted for chemotherapy regimen, age at surgery (continuous for symptom domains, quartiles for individual symptoms), body mass index (BMI) category (<18.5, 18.5–25, 25–30, >30 kg/m^2^), race/ethnicity (non-Hispanic white, non-Hispanic Black, Hispanic, Other), type of breast surgery (lumpectomy, mastectomy), and hormonal therapy (yes vs no; only in the subacute period) as well as baseline domain-level TI and baseline symptom, respectively.

In PIM, the probability that a cumulative TI value for one group is greater than or equal to the value for another group was estimated along with the corresponding 95% confidence interval. Confidence intervals and *p*-values for treatment comparisons were adjusted for multiple tests using the Bonferroni and Holm^25^ procedures, respectively. Analyses were performed using R package version 4.0.5^26^ with two-sided tests at a significance level of 0.05.

### Reporting summary

Further information on research design is available in the Nature Research Reporting Summary linked to this article.

## Supplementary information

Supplementary information

Reporting Summary

## Data Availability

Individual participant data that underlie the results reported in this article will be made available after de-identification (text, tables, figures, and appendices). The study protocol will also be available. Data will be made available beginning 9 months after publication. Investigators who wish to use the data will have to follow standard NCI NCTC/NCORP Data Archive guidelines for obtaining data, which includes submitting a brief research plan and obtaining a data use agreement between the Data Archive and the investigator’s institution (https://nctn-data-archive.nci.nih.gov/about-us).
