# Supplementary material for: Toxicity Index, patient-reported outcomes, and persistence of breast cancer chemotherapy-associated side effects in NRG Oncology/NSABP B-30
Source: NPJ Breast Cancer. 2022 Nov 19;8:123. doi: 10.1038/s41523-022-00489-9 (PMC9675799; doi:10.1038/s41523-022-00489-9)
Supplement: Supplementary file 1 — Supplementary information [file 41523_2022_489_MOESM1_ESM.pdf]

Supplementary Files

Toxicity Index, patient-reported outcomes, and persistence of breast cancer chemotherapy-associated side effects in NRG Oncology/NSABP B-30

N. L. Henry, S. Kim, R. D. Hays, M. A. Diniz, M. Tighiouart, G. Gresham, M. Luu, R. S. Cecchini, G. Yothers, A. Rogatko, P. A. Ganz

Contents:

Supplementary Figure 1. Visual depiction of data timepoints that are included in the acute and subacute periods.

Supplementary Table 1. Comparison of baseline patient characteristics between participants with and without data in the subacute period.

Supplementary Table 2. Number of participants with one or more non-missing values for patient reported outcomes data, by cycle and study arm.

Supplementary Table 3. Multivariable Probabilistic Index Models (PIM) for the cumulative Toxicity Index (TI) for the pain domain during the acute and subacute time periods.

Supplementary Table 4. Multivariable PIM for the cumulative TI for pain domain individual items during the acute time period.

Supplementary Table 5. Multivariable PIM for the cumulative TI for pain domain individual items during the subacute time period.

Supplementary Table 6. Multivariable PIM for the cumulative TI for the cognitive domain during the acute and subacute time periods.

Supplementary Table 7. Multivariable PIM for the cumulative TI for cognitive domain individual items during the acute time period.

Supplementary Table 8. Multivariable PIM for the cumulative TI for cognitive domain individual items during the subacute time period.

Supplementary Table 9. Multivariable PIM for the cumulative TI for the vasomotor domain during the acute and subacute time periods.

Supplementary Table 10. Multivariable PIM for the cumulative TI for vasomotor domain individual items during the acute time period.

Supplementary Table 11. Multivariable PIM for the cumulative TI for vasomotor domain individual items during the subacute time period.

Supplementary Table 12. Multivariable PIM for the cumulative TI for the vaginal domain during the acute and subacute time periods.

Supplementary Table 13. Multivariable PIM for the cumulative TI for vaginal domain individual items during the acute time period.

Supplementary Table 14. Multivariable PIM for the cumulative TI for vaginal domain individual items during the subacute time period.

Supplementary Figure 1

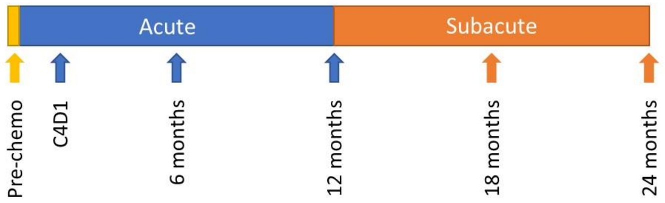

Visual depiction of data timepoints that are included in the acute (blue) and subacute (orange) periods. Patient-reported outcomes were completed at the 5 timepoints designated in blue and orange, and reflected the symptoms in the 7 days prior to each assessment.

Supplementary Table 1. Comparison of baseline patient characteristics between participants with and without data in the subacute period. Bolded p values reflect those that are <0.05. NH: Non-Hispanic

Variable	All patients (*N*=2111)	Those with data in subacute period (*N*=1802)	Those without data in subacute period (*N*=309)	*P*-value
Age at surgery (Years), Median (IQR)	49 (43 - 57)	50 (44 - 57)	48 (42 - 56)	0.097
Age at surgery (Years)				0.292
[24, 43]	462 (21.89)	384 (21.31)	78 (25.24)	
[43, 50]	594 (28.14)	504 (27.97)	90 (29.13)	
[50, 57]	505 (23.92)	441 (24.47)	64 (20.71)	
[57, 80]	550 (26.05)	473 (26.25)	77 (24.92)	
Race/Ethnicity [1]				0.079
NH White	1817 (86.07)	1564 (86.79)	253 (81.88)	
Hispanic	59 (2.79)	44 (2.44)	15 (4.85)	
NH Black	174 (8.24)	142 (7.88)	32 (10.36)	
Other	60 (2.84)	51 (2.83)	9 (2.91)	
Unknown	1 (0.05)	1 (0.06)	0 (0)	
Body mass index (BMI; kg/m2)				0.913
Underweight BMI < 18.5	24 (1.14)	20 (1.11)	4 (1.29)	
Normal weight 18.5 ≤ BMI < 25	771 (36.52)	663 (36.79)	108 (34.95)	
Overweight 25 ≤ BMI < 30	682 (32.31)	578 (32.08)	104 (33.66)	
Obesity BMI ≥ 30	634 (30.03)	541 (30.02)	93 (30.1)	
Type of surgery				0.825
Lumpectomy	1078 (51.07)	922 (51.17)	156 (50.49)	
Mastectomy	1033 (48.93)	880 (48.83)	153 (49.51)	
Hormonal therapy				**<0.001**
Yes	1672 (79.2)	1455 (80.74)	217 (70.23)	
No	439 (20.8)	347 (19.26)	92 (29.77)	

Data are presented as number of patients (column %) or median (IQR, interquartile range).
P-value is calculated by Wilcoxon rank-sum test for the continuous variable and chi-square test for categorical variables as appropriate.
[1] Unknown race/ethnicity is excluded from analyses.

Supplementary Table 2. Number of participants with one or more non-missing values for patient reported outcomes data, by cycle and study arm A: doxorubicin; C: cyclophosphamide; QOL: quality of life; T: docetaxel

	**Total**	**AC -> T**	**AT**	**ATC**
Participants included in the QOL substudy	2156	717	718	721
Participants with follow-up questionnaires	2111	707	702	702
Baseline	2111 (100)	707 (100)	702 (100)	702 (100)
Day 1 of Cycle 4	1890 (89.5)	617 (87.3)	642 (91.5)	631 (89.9)
6-month follow-up	1764 (83.6)	595 (84.2)	587 (83.6)	582 (82.9)
12-month follow-up	1686 (79.9)	558 (78.9)	555 (79.1)	573 (81.6)
18-month follow-up	1551 (73.5)	523 (74.0)	507 (72.2)	521 (74.2)
24-month follow-up	1571 (74.4)	521 (73.7)	523 (74.5)	527 (75.1)
Data are presented as number of participants (%).				

Supplementary Table 3. Multivariable Probabilistic Index Models for the cumulative Toxicity Index (TI) for the pain domain during the acute and subacute time periods Bolded p values reflect those that are <0.05. A: doxorubicin, BMI: body-mass index, C: cyclophosphamide, CI: confidence interval, NH: non-Hispanic, T: docetaxel.

		Acute Period (*N*=2075)		Subacute Period (*N*=1791)	
**Variable**	**Comparison A < B [1]**	**Probability (95% CI) [2]**	***P*-value [2]**	**Probability (95% CI) [2]**	***P*-value [3]**
**Treatment [4]**	AT < ATC	0.505 (0.468, 0.541)	0.768	0.512 (0.471, 0.552)	0.489
	AT < AC -> T	0.637 (0.599, 0.673)	**<0.001**	0.572 (0.531, 0.613)	**<0.001**
	ATC < AC -> T	0.633 (0.595, 0.669)	**<0.001**	0.561 (0.519, 0.602)	**0.001**
**TI at Baseline**	1-unit increment	0.598 (0.587, 0.609)	**<0.001**	0.599 (0.587, 0.612)	**<0.001**
**Age at surgery (Years)**	1-year increment	0.501 (0.499, 0.502)	0.351	0.503 (0.502, 0.505)	**<0.001**
**BMI (kg/m^2)**	Obesity (BMI >= 30) < Underweight (BMI < 18.5)	0.48 (0.341, 0.623)	0.787	0.374 (0.236, 0.537)	0.128
	Obesity (BMI >= 30) < Normal (18.5 <= BMI < 25)	0.452 (0.42, 0.484)	**0.004**	0.406 (0.373, 0.441)	**<0.001**
	Obesity (BMI >= 30) < Overweight (25 <= BMI < 30)	0.475 (0.442, 0.507)	0.127	0.452 (0.418, 0.487)	**0.007**
**Race/Ethnicity [5]**	NH White < NH Black	0.572 (0.52, 0.623)	**0.007**	0.537 (0.478, 0.595)	0.224
	NH White < Hispanic	0.543 (0.464, 0.621)	0.287	0.458 (0.359, 0.56)	0.421
	NH White < Other	0.512 (0.433, 0.59)	0.773	0.532 (0.446, 0.617)	0.462
**Type of surgery**	Lumpectomy < Mastectomy	0.503 (0.477, 0.529)	0.820	0.537 (0.509, 0.564)	**0.009**
**Endocrine therapy**	No < Yes	N/A		0.51 (0.474, 0.546)	0.585

[1] Comparison A < B denotes the probability that the cumulative TI for B is higher than that for A

[2] Probability of 0.5 indicates no difference between comparisons (i.e., A=B)

[3] *P* values are calculated using the Wald statistic

[4] CI are adjusted for multiple tests for treatment using the Bonferroni procedure; *p*-values are adjusted for multiple tests for treatment using the Holm procedure

[5] Unknown race/ethnicity was excluded from analysis

Supplementary Table 4. Multivariable Probabilistic Index Models for the cumulative Toxicity Index (TI) for pain domain individual items during the acute time period Bolded p values reflect those that are <0.05. A: doxorubicin, C: cyclophosphamide, CI: confidence interval, NH: non-Hispanic, T: docetaxel.

		Numbness (*N*=2067)		General Aches and Pains (*N*=2066)		Joint Pains (*N*=2064)		Swelling of Hands or Feet (*N*=2066)		Muscle Stiffness *N*=2058)	
**Variable**	**Comparison A < B [1]**	**Probability (95% CI) [2]**	***P*-value [3]**	**Probability (95% CI) [2]**	***P*-value [3]**	**Probability (95% CI) [2]**	***P*-value [3]**	**Probability (95% CI) [2]**	***P*-value [3]**	**Probability (95% CI) [2]**	***P*-value [3]**
**Treatment [4]**	AT < ATC	0.533 (0.499, 0.567)	**0.022**	0.502 (0.465, 0.539)	0.899	0.523 (0.486, 0.56)	0.263	0.508 (0.472, 0.543)	0.611	0.502 (0.465, 0.54)	0.877
	AT < AC -> T	0.691 (0.657, 0.722)	**<0.001**	0.556 (0.517, 0.593)	**0.002**	0.544 (0.506, 0.582)	**0.018**	0.585 (0.549, 0.621)	**<0.001**	0.587 (0.55, 0.623)	**<0.001**
	ATC < AC -> T	0.662 (0.626, 0.696)	**<0.001**	0.554 (0.515, 0.591)	**0.002**	0.521 (0.482, 0.559)	0.263	0.578 (0.541, 0.614)	**<0.001**	0.585 (0.548, 0.621)	**<0.001**
**Symptom at Baseline**	1-unit increment	0.598 (0.575, 0.62)	**<0.001**	0.636 (0.62, 0.65)	**<0.001**	0.658 (0.641, 0.674)	**<0.001**	0.641 (0.616, 0.665)	**<0.001**	0.609 (0.593, 0.624)	**<0.001**
**Age at surgery (Years)**	[57, 80] < [24, 43)	0.465 (0.430, 0.500)	0.051	0.488 (0.45, 0.525)	0.522	0.487 (0.45, 0.524)	0.478	0.496 (0.46, 0.533)	0.845	0.484 (0.448, 0.521)	0.395
	[57, 80] < [43, 50)	0.472 (0.439, 0.505)	0.097	0.517 (0.483, 0.551)	0.330	0.524 (0.489, 0.559)	0.177	0.508 (0.475, 0.541)	0.636	0.494 (0.46, 0.528)	0.737
	[57, 80] < [50, 57)	0.493 (0.458, 0.527)	0.685	0.513 (0.477, 0.549)	0.477	0.531 (0.495, 0.566)	0.092	0.525 (0.491, 0.559)	0.156	0.516 (0.482, 0.551)	0.358
**BMI (kg/m^2)**	Obesity < Underweight	0.476 (0.358, 0.597)	0.700	0.475 (0.345, 0.609)	0.722	0.448 (0.329, 0.574)	0.420	0.333 (0.25, 0.428)	**0.001**	0.505 (0.383, 0.627)	0.933
	Obesity < Normal	0.458 (0.429, 0.489)	**0.007**	0.456 (0.425, 0.488)	**0.007**	0.469 (0.437, 0.502)	0.064	0.387 (0.358, 0.418)	**<0.001**	0.473 (0.442, 0.505)	0.096
	Obesity < Overweight	0.48 (0.449, 0.512)	0.217	0.472 (0.439, 0.505)	0.092	0.484 (0.452, 0.517)	0.350	0.437 (0.406, 0.468)	**<0.001**	0.475 (0.443, 0.507)	0.120
**Race/Ethnicity [5]**	NH White < NH Black	0.585 (0.538, 0.63)	**<0.001**	0.541 (0.492, 0.59)	0.101	0.522 (0.472, 0.573)	0.387	0.524 (0.473, 0.574)	0.363	0.535 (0.486, 0.583)	0.159
	NH White < Hispanic	0.519 (0.451, 0.586)	0.586	0.534 (0.446, 0.621)	0.451	0.562 (0.476, 0.644)	0.159	0.458 (0.386, 0.532)	0.265	0.457 (0.379, 0.537)	0.291
	NH White < Other	0.556 (0.484, 0.627)	0.128	0.474 (0.392, 0.556)	0.530	0.488 (0.41, 0.566)	0.759	0.467 (0.396, 0.538)	0.361	0.447 (0.376, 0.52)	0.156
**Type of surgery**	Lumpectomy < Mastectomy	0.517 (0.493, 0.541)	0.162	0.493 (0.467, 0.518)	0.585	0.502 (0.476, 0.527)	0.891	0.501 (0.477, 0.525)	0.942	0.496 (0.471, 0.521)	0.742

[1] Comparison A < B denotes the probability that the cumulative TI for B is higher than that for A

[2] Probability of 0.5 indicates no difference between comparisons (i.e., A=B)

[3] *P* values are calculated using the Wald statistic

[4] CI are adjusted for multiple tests for treatment using the Bonferroni procedure; *p*-values are adjusted for multiple tests for treatment using the Holm procedure

[5] Unknown race/ethnicity was excluded from analysis

Supplementary Table 5. Multivariable Probabilistic Index Models for the cumulative Toxicity Index (TI) for pain domain individual items during the subacute time period Bolded p values reflect those that are <0.05. A: doxorubicin, C: cyclophosphamide, CI: confidence interval, NH: non-Hispanic, T: docetaxel.

		Numbness (*N*=1777)		General Aches and Pains (*N*=1780)		Joint Pains (*N*=1783)		Swelling of Hands or Feet (*N*=1782)		Muscle Stiffness (*N*=1776)	
**Variable**	**Comparison A < B [1]**	**Probability (95% CI) [2]**	***P*-value [3]**	**Probability (95% CI) [2]**	***P*-value [3]**	**Probability (95% CI) [2]**	***P*-value [3]**	**Probability (95% CI) [2]**	***P*-value [3]**	**Probability (95% CI) [2]**	***P*-value [3]**
**Treatment [4]**	AT < ATC	0.519 (0.483, 0.555)	0.207	0.506 (0.466, 0.547)	0.910	0.528 (0.488, 0.567)	0.158	0.504 (0.467, 0.542)	0.780	0.522 (0.482, 0.562)	0.374
	AT < AC -> T	0.598 (0.561, 0.635)	**<0.001**	0.519 (0.478, 0.56)	0.815	0.558 (0.517, 0.598)	**0.002**	0.525 (0.487, 0.563)	0.352	0.542 (0.502, 0.582)	**0.036**
	ATC < AC -> T	0.58 (0.541, 0.618)	**<0.001**	0.513 (0.472, 0.553)	0.910	0.531 (0.489, 0.572)	0.158	0.52 (0.482, 0.559)	0.405	0.52 (0.48, 0.56)	0.374
**Symptom at baseline**	1-unit increment	0.596 (0.571, 0.62)	**<0.001**	0.636 (0.619, 0.652)	**<0.001**	0.642 (0.624, 0.66)	**<0.001**	0.62 (0.594, 0.646)	**<0.001**	0.591 (0.574, 0.607)	**<0.001**
**Age at surgery (Years)**	[57, 80] < [24, 43)	0.443 (0.406, 0.48)	**0.003**	0.434 (0.395, 0.473)	**0.001**	0.426 (0.387, 0.467)	**<0.001**	0.483 (0.446, 0.52)	0.361	0.408 (0.37, 0.446)	**<0.001**
	[57, 80] < [43, 50)	0.459 (0.424, 0.493)	**0.019**	0.479 (0.442, 0.516)	0.274	0.484 (0.447, 0.522)	0.412	0.499 (0.464, 0.534)	0.946	0.469 (0.433, 0.505)	0.090
	[57, 80] < [50, 57)	0.48 (0.444, 0.516)	0.270	0.501 (0.463, 0.539)	0.965	0.512 (0.474, 0.549)	0.540	0.538 (0.502, 0.573)	**0.036**	0.526 (0.49, 0.563)	0.159
**BMI (kg/m^2)**	Obesity < Underweight	0.475 (0.352, 0.601)	0.697	0.356 (0.238, 0.494)	**0.041**	0.428 (0.287, 0.582)	0.359	0.385 (0.28, 0.503)	0.056	0.386 (0.252, 0.539)	0.142
	Obesity < Normal	0.414 (0.383, 0.446)	**<0.001**	0.422 (0.388, 0.456)	**<0.001**	0.437 (0.403, 0.471)	**<0.001**	0.396 (0.365, 0.428)	**<0.001**	0.413 (0.38, 0.446)	**<0.001**
	Obesity < Overweight	0.471 (0.438, 0.504)	0.082	0.459 (0.424, 0.493)	**0.020**	0.471 (0.436, 0.506)	0.099	0.442 (0.409, 0.475)	**0.001**	0.449 (0.416, 0.483)	**0.003**
**Race/Ethnicity [5]**	NH White < NH Black	0.509 (0.461, 0.557)	0.718	0.536 (0.48, 0.591)	0.212	0.493 (0.436, 0.549)	0.797	0.521 (0.468, 0.573)	0.441	0.505 (0.452, 0.559)	0.841
	NH White < Hispanic	0.512 (0.432, 0.591)	0.769	0.476 (0.379, 0.575)	0.632	0.488 (0.387, 0.59)	0.818	0.447 (0.37, 0.525)	0.183	0.407 (0.324, 0.495)	**0.038**
	NH White < Other	0.518 (0.436, 0.6)	0.664	0.511 (0.415, 0.606)	0.829	0.488 (0.404, 0.573)	0.786	0.444 (0.378, 0.512)	0.106	0.446 (0.374, 0.519)	0.149
**Type of mastectomy**	Lumpectomy < Mastectomy	0.549 (0.524, 0.574)	**<0.001**	0.513 (0.485, 0.54)	0.356	0.533 (0.506, 0.56)	**0.018**	0.527 (0.501, 0.552)	**0.039**	0.521 (0.494, 0.548)	0.134

**Endocrine therapy**	No < Yes	0.493 (0.461, 0.525)	0.683	0.491 (0.457, 0.525)	0.604	0.522 (0.488, 0.555)	0.209	0.533 (0.501, 0.564)	**0.043**	0.526 (0.492, 0.56)	0.128

[1] Comparison A < B denotes the probability that the cumulative TI for B is higher than that for A

[2] Probability of 0.5 indicates no difference between comparisons (i.e., A=B)

[3] *P* values are calculated using the Wald statistic

[4] CI are adjusted for multiple tests for treatment using the Bonferroni procedure; *p*-values are adjusted for multiple tests for treatment using the Holm procedure

[5] Unknown race/ethnicity was excluded from analysis

Supplementary Table 6. Multivariable Probabilistic Index Models for the cumulative Toxicity Index (TI) for cognitive domain during the acute and subacute time periods Bolded p values reflect those that are <0.05. A: doxorubicin, BMI: body-mass index, C: cyclophosphamide, CI: confidence interval, NH: non-Hispanic, T: docetaxel.

		Acute Period (*N*=2078)		Subacute Period (*N*=1793)	
**Variable**	**Comparison A < B [1]**	**Probability (95% CI) [2]**	***P*-value [3]**	**Probability (95% CI) [2]**	***P*-value [3]**
**Treatment [4]**	AT < ATC	0.51 (0.471, 0.548)	1.000	0.52 (0.48, 0.56)	0.694
	AT < AC -> T	0.516 (0.477, 0.555)	0.964	0.517 (0.475, 0.559)	0.694
	ATC < AC -> T	0.507 (0.468, 0.545)	1.000	0.497 (0.456, 0.539)	0.866
**TI at Baseline**	1-unit increment	0.627 (0.615, 0.639)	**<0.001**	0.609 (0.596, 0.621)	**<0.001**
**Age at surgery (Years)**	1-year increment	0.497 (0.495, 0.498)	**<0.001**	0.499 (0.497, 0.5)	0.118
**BMI (kg/m^2)**	Obesity (BMI >= 30) < Underweight (BMI < 18.5)	0.443 (0.319, 0.576)	0.402	0.446 (0.319, 0.58)	0.428
	Obesity (BMI >= 30) < Normal (18.5 <= BMI < 25)	0.487 (0.455, 0.519)	0.426	0.48 (0.446, 0.515)	0.261
	Obesity (BMI >= 30) < Overweight (25 <= BMI < 30)	0.497 (0.463, 0.53)	0.853	0.5 (0.465, 0.535)	0.989
**Race/Ethnicity [5]**	NH White < NH Black	0.523 (0.472, 0.573)	0.374	0.524 (0.469, 0.579)	0.397
	NH White < Hispanic	0.547 (0.457, 0.633)	0.310	0.467 (0.367, 0.57)	0.530
	NH White < Other	0.435 (0.352, 0.521)	0.138	0.539 (0.444, 0.631)	0.424
**Type of surgery**	Lumpectomy < Mastectomy	0.507 (0.481, 0.533)	0.581	0.505 (0.477, 0.532)	0.733
**Endocrine therapy**	No < Yes	N/A		0.52 (0.485, 0.556)	0.263

[1] Comparison A < B denotes the probability that the cumulative TI for B is higher than that for A

[2] Probability of 0.5 indicates no difference between comparisons (i.e., A=B)

[3] *P* values are calculated using the Wald statistic

[4] CI are adjusted for multiple tests for treatment using the Bonferroni procedure; *p*-values are adjusted for multiple tests for treatment using the Holm procedure

[5] Unknown race/ethnicity was excluded from analysis

Supplementary Table 7. Multivariable Probabilistic Index Models for the cumulative Toxicity Index (TI) for cognition domain individual items during the acute time period Bolded p values reflect those that are <0.05. A: doxorubicin, C: cyclophosphamide, CI: confidence interval, NH: non-Hispanic, T: docetaxel.

		**Mood Swings (*N*=2070)**		**Forgetfulness (*N*=2072)**		**Difficulty Concentrating (*N*=2072)**	
**Variable**	**Comparison A < B [1]**	**Probability (95% CI) [2]**	***P*-value [3]**	**Probability (95% CI) [2]**	***P*-value [3]**	**Probability (95% CI) [2]**	***P*-value [3]**
**Treatment [4]**	AT < ATC	0.508 (0.470, 0.546)	0.812	0.504 (0.466, 0.542)	1.000	0.485 (0.447, 0.522)	0.656
	AT < AC -> T	0.521 (0.484, 0.559)	0.523	0.511 (0.473, 0.549)	1.000	0.507 (0.469, 0.546)	0.656
	ATC < AC -> T	0.513 (0.475, 0.551)	0.812	0.508 (0.470, 0.545)	1.000	0.523 (0.485, 0.560)	0.445
**Symptom at Baseline**	1-unit increment	0.643 (0.629, 0.657)	**<0.001**	0.662 (0.646, 0.678)	**<0.001**	0.660 (0.644, 0.676)	**<0.001**
**Age at surgery (Years)**	[57, 80] < [24, 43)	0.596 (0.560, 0.632)	**<0.001**	0.547 (0.509, 0.585)	**0.015**	0.537 (0.499, 0.574)	0.054
	[57, 80] < [43, 50)	0.570 (0.536, 0.604)	**<0.001**	0.549 (0.515, 0.582)	**0.005**	0.543 (0.509, 0.577)	**0.013**
	[57, 80] < [50, 57)	0.515 (0.480, 0.551)	0.393	0.531 (0.496, 0.566)	0.083	0.546 (0.511, 0.581)	**0.010**
**BMI (kg/m²)**	Obesity < Underweight	0.444 (0.313, 0.583)	0.427	0.536 (0.418, 0.651)	0.549	0.450 (0.327, 0.580)	0.451
	Obesity < Normal	0.471 (0.439, 0.503)	0.073	0.506 (0.474, 0.538)	0.711	0.505 (0.473, 0.537)	0.748
	Obesity < Overweight	0.483 (0.45, 0.515)	0.299	0.512 (0.479, 0.544)	0.487	0.512 (0.479, 0.545)	0.465
**Race/Ethnicity [5]**	NH White < NH Black	0.552 (0.503, 0.6)	**0.038**	0.479 (0.430, 0.528)	0.397	0.455 (0.405, 0.506)	0.081
	NH White < Hispanic	0.504 (0.428, 0.581)	0.910	0.518 (0.435, 0.600)	0.667	0.527 (0.442, 0.611)	0.536
	NH White < Other	0.492 (0.414, 0.57)	0.840	0.436 (0.362, 0.513)	0.103	0.440 (0.366, 0.518)	0.13
**Type of surgery**	Lumpectomy < Mastectomy	0.497 (0.472, 0.523)	0.822	0.499 (0.474, 0.525)	0.959	0.510 (0.484, 0.535)	0.451

[1] Comparison A < B denotes the probability that the cumulative TI for B is higher than that for A
[2] Probability of 0.5 indicates no difference between comparisons (i.e., A=B)
[3] *P* values are calculated using the Wald statistic
[4] CI are adjusted for multiple tests for treatment using the Bonferroni procedure; *p*-values are adjusted for multiple tests for treatment using the Holm procedure
[5] Unknown race/ethnicity was excluded from analysis

Supplementary Table 8. Multivariable Probabilistic Index Models for the cumulative Toxicity Index (TI) for cognition domain individual items during the subacute time period Bolded p values reflect those that are <0.05. A: doxorubicin, C: cyclophosphamide, CI: confidence interval, NH: non-Hispanic, T: docetaxel.

		**Mood Swings (*N*=1784)**		**Forgetfulness (*N*=1786)**		**Difficulty Concentrating (*N*=1790)**	
**Variable**	**Comparison A < B [1]**	**Probability (95% CI) [2]**	***P*-value [3]**	**Probability (95% CI) [2]**	***P*-value [3]**	**Probability (95% CI) [2]**	***P*-value [3]**
**Treatment [4]**	AT < ATC	0.518 (0.479, 0.557)	0.793	0.526 (0.487, 0.565)	0.346	0.503 (0.465, 0.542)	0.898
	AT < AC -> T	0.516 (0.476, 0.555)	0.793	0.521 (0.48, 0.562)	0.433	0.517 (0.477, 0.558)	0.898
	ATC < AC -> T	0.498 (0.458, 0.537)	0.886	0.495 (0.455, 0.536)	0.784	0.514 (0.474, 0.554)	0.898
**Symptom at baseline**	1-unit increment	0.601 (0.586, 0.617)	**<0.001**	0.633 (0.617, 0.649)	**<0.001**	0.646 (0.63, 0.662)	**<0.001**
**Age at surgery (Years)**	[57, 80] < [24, 43)	0.576 (0.537, 0.613)	**<0.001**	0.478 (0.439, 0.518)	0.279	0.494 (0.456, 0.532)	0.760
	[57, 80] < [43, 50)	0.544 (0.509, 0.579)	**0.015**	0.506 (0.47, 0.542)	0.748	0.535 (0.499, 0.57)	0.056
	[57, 80] < [50, 57)	0.525 (0.489, 0.561)	0.172	0.512 (0.475, 0.549)	0.514	0.525 (0.488, 0.561)	0.187
**BMI (kg/m^2)**	Obesity < Underweight	0.5 (0.362, 0.639)	0.998	0.452 (0.328, 0.582)	0.468	0.444 (0.341, 0.553)	0.316
	Obesity < Normal	0.474 (0.441, 0.507)	0.124	0.5 (0.466, 0.534)	1.000	0.487 (0.453, 0.521)	0.449
	Obesity < Overweight	0.497 (0.463, 0.53)	0.845	0.517 (0.483, 0.55)	0.337	0.496 (0.462, 0.531)	0.836
**Race/Ethnicity[5]**	NH White < NH Black	0.527 (0.476, 0.579)	0.297	0.508 (0.454, 0.561)	0.780	0.49 (0.438, 0.542)	0.708
	NH White < Hispanic	0.497 (0.409, 0.584)	0.941	0.445 (0.357, 0.536)	0.237	0.458 (0.374, 0.546)	0.351
	NH White < Other	0.523 (0.438, 0.607)	0.596	0.513 (0.424, 0.6)	0.781	0.509 (0.439, 0.579)	0.804
**Type of surgery**	Lumpectomy < Mastectomy	0.499 (0.473, 0.525)	0.935	0.502 (0.475, 0.529)	0.866	0.505 (0.479, 0.532)	0.706
**Endocrine therapy**	No < Yes	0.482 (0.449, 0.516)	0.310	0.524 (0.489, 0.559)	0.171	0.521 (0.488, 0.555)	0.208

[1] Comparison A < B denotes the probability that the cumulative TI for B is higher than that for A
[2] Probability of 0.5 indicates no difference between comparisons (i.e., A=B)
[3] *P* values are calculated using the Wald statistic
[4] CI are adjusted for multiple tests for treatment using the Bonferroni procedure; *p*-values are adjusted for multiple tests for treatment using the Holm procedure
[5] Unknown race/ethnicity was excluded from analysis

Supplementary Table 9. Multivariable Probabilistic Index Models for the cumulative Toxicity Index (TI) for the vasomotor domain during the acute and subacute time periods Bolded p values reflect those that are <0.05. A: doxorubicin, BMI: body-mass index, C: cyclophosphamide, CI: confidence interval, NH: non-Hispanic, T: docetaxel.

		Acute Period (*N*=2075)		Subacute Period (*N*=1791)	
**Variable**	**Comparison A < B [1]**	**Probability (95% CI) [2]**	***P*-value [3]**	**Probability (95% CI) [2]**	***P*-value [3]**
**Treatment [4]**	AT < ATC	0.568 (0.53, 0.606)	**<0.001**	0.577 (0.537, 0.616)	**<0.001**
	AT < AC -> T	0.508 (0.469, 0.547)	0.617	0.545 (0.503, 0.585)	**0.019**
	ATC < AC -> T	0.439 (0.402, 0.478)	**<0.001**	0.467 (0.426, 0.508)	0.057
**TI at Baseline**	1-unit increment	0.595 (0.585, 0.604)	**<0.001**	0.584 (0.573, 0.594)	**<0.001**
**Age at surgery (Years)**	1-year increment	0.491 (0.489, 0.492)	**<0.001**	0.497 (0.496, 0.499)	**0.001**
**BMI (kg/m^2)**	Obesity (BMI >= 30) < Underweight (BMI < 18.5)	0.442 (0.345, 0.544)	0.262	0.317 (0.213, 0.443)	**0.005**
	Obesity (BMI >= 30) < Normal (18.5 <= BMI < 25)	0.514 (0.482, 0.546)	0.398	0.498 (0.464, 0.532)	0.917
	Obesity (BMI >= 30) < Overweight (25 <= BMI < 30)	0.54 (0.507, 0.572)	**0.018**	0.528 (0.493, 0.562)	0.117
**Race/Ethnicity [5]**	NH White < NH Black	0.494 (0.443, 0.546)	0.826	0.519 (0.462, 0.575)	0.522
	NH White < Hispanic	0.444 (0.362, 0.529)	0.197	0.412 (0.315, 0.516)	0.095
	NH White < Other	0.445 (0.368, 0.525)	0.174	0.517 (0.433, 0.599)	0.697
**Type of surgery**	Lumpectomy < Mastectomy	0.504 (0.479, 0.53)	0.735	0.515 (0.487, 0.542)	0.302
**Endocrine therapy**	No < Yes	N/A		0.656 (0.622, 0.688)	**<0.001**

[1] Comparison A < B denotes the probability that the cumulative TI for B is higher than that for A
[2] Probability of 0.5 indicates no difference between comparisons (i.e., A=B)
[3] *P* values are calculated using the Wald statistic
[4] CI are adjusted for multiple tests for treatment using the Bonferroni procedure; *p*-values are adjusted for multiple tests for treatment using the Holm procedure
[5] Unknown race/ethnicity was excluded from analysis

Supplementary Table 10. Multivariable Probabilistic Index Models for the cumulative Toxicity Index (TI) for vasomotor domain individual items during the acute time period Bolded p values reflect those that are <0.05. A: doxorubicin, C: cyclophosphamide, CI: confidence interval, NH: non-Hispanic, T: docetaxel.

		**Hot Flashes (*N*=2072)**		**Night Sweats (*N*=2069)**	
**Variable**	**Comparison A < B [1]**	**Probability (95% CI) [2]**	***P*-value [3]**	**Probability (95% CI) [2]**	***P*-value [3]**
**Treatment [4]**	AT < ATC	0.573 (0.535, 0.61)	**<0.001**	0.571 (0.533, 0.608)	**<0.001**
	AT < AC -> T	0.515 (0.476, 0.553)	0.354	0.523 (0.484, 0.561)	0.160
	ATC < AC -> T	0.442 (0.404, 0.479)	**<0.001**	0.451 (0.414, 0.489)	**0.004**
**Symptom at Baseline**	1-unit increment	0.602 (0.59, 0.614)	**<0.001**	0.623 (0.609, 0.637)	**<0.001**
**Age at surgery (Years)**	[57, 80] < [24, 43)	0.673 (0.638, 0.706)	**<0.001**	0.68 (0.646, 0.713)	**<0.001**
	[57, 80] < [43, 50)	0.722 (0.692, 0.75)	**<0.001**	0.72 (0.691, 0.748)	**<0.001**
	[57, 80] < [50, 57)	0.636 (0.603, 0.669)	**<0.001**	0.639 (0.606, 0.671)	**<0.001**
**BMI (kg/m^2)**	Obesity < Underweight	0.501 (0.378, 0.624)	0.989	0.463 (0.363, 0.566)	0.483
	Obesity < Normal	0.518 (0.485, 0.55)	0.279	0.532 (0.5, 0.564)	0.050
	Obesity < Overweight	0.541 (0.508, 0.573)	**0.015**	0.537 (0.504, 0.57)	**0.026**
**Race/Ethnicity [5]**	NH White < NH Black	0.503 (0.452, 0.554)	0.894	0.493 (0.443, 0.543)	0.780
	NH White < Hispanic	0.469 (0.388, 0.553)	0.474	0.442 (0.365, 0.521)	0.150
	NH White < Other	0.444 (0.364, 0.527)	0.188	0.415 (0.347, 0.486)	**0.019**
**Type of surgery**	Lumpectomy < Mastectomy	0.502 (0.477, 0.528)	0.857	0.503 (0.477, 0.529)	0.821

[1] Comparison A < B denotes the probability that the cumulative TI for B is higher than that for A
[2] Probability of 0.5 indicates no difference between comparisons (i.e., A=B)
[3] *P* values are calculated using the Wald statistic
[4] CI are adjusted for multiple tests for treatment using the Bonferroni procedure; *p*-values are adjusted for multiple tests for treatment using the Holm procedure
[5] Unknown race/ethnicity was excluded from analysis

Supplementary Table 11. Multivariable Probabilistic Index Models for the cumulative Toxicity Index (TI) for vasomotor domain individual items during the subacute time period Bolded p values reflect those that are <0.05. A: doxorubicin, C: cyclophosphamide, CI: confidence interval, NH: non-Hispanic, T: docetaxel.

		Hot Flashes (*N*=1787)		Night Sweats (*N*=1786)	
**Variable**	**Comparison A < B [1]**	**Probability (95% CI) [2]**	***P*-value [3]**	**Probability (95% CI) [2]**	***P*-value [3]**
**Treatment [4]**	AT < ATC	0.582 (0.542, 0.621)	**<0.001**	0.56 (0.521, 0.599)	**0.001**
	AT < AC -> T	0.559 (0.518, 0.6)	**0.001**	0.536 (0.496, 0.575)	0.059
	ATC < AC -> T	0.477 (0.436, 0.518)	0.183	0.476 (0.436, 0.516)	0.145
**Symptom at baseline**	1-unit increment	0.592 (0.579, 0.604)	**<0.001**	0.602 (0.588, 0.617)	**<0.001**
**Age at surgery (Years)**	[57, 80] < [24, 43)	0.515 (0.473, 0.556)	0.483	0.542 (0.503, 0.58)	**0.034**
	[57, 80] < [43, 50)	0.648 (0.613, 0.682)	**<0.001**	0.641 (0.606, 0.673)	**<0.001**
	[57, 80] < [50, 57)	0.615 (0.578, 0.651)	**<0.001**	0.635 (0.6, 0.669)	**<0.001**
**BMI (kg/m^2)**	Obesity < Underweight	0.378 (0.25, 0.526)	0.106	0.303 (0.203, 0.426)	**0.002**
	Obesity < Normal	0.509 (0.475, 0.543)	0.617	0.515 (0.481, 0.549)	0.386
	Obesity < Overweight	0.534 (0.499, 0.568)	0.056	0.523 (0.489, 0.557)	0.185
**Race/Ethnicity [5]**	NH White < NH Black	0.522 (0.467, 0.576)	0.428	0.523 (0.471, 0.575)	0.384
	NH White < Hispanic	0.439 (0.347, 0.536)	0.215	0.45 (0.358, 0.546)	0.307
	NH White < Other	0.499 (0.414, 0.584)	0.982	0.454 (0.378, 0.532)	0.245
**Type of surgery**	Lumpectomy < Mastectomy	0.51 (0.482, 0.537)	0.497	0.518 (0.491, 0.545)	0.190
**Endocrine therapy**	No < Yes	0.66 (0.628, 0.691)	**<0.001**	0.625 (0.594, 0.656)	**<0.001**

[1] Comparison A < B denotes the probability that the cumulative TI for B is higher than that for A
[2] Probability of 0.5 indicates no difference between comparisons (i.e., A=B)
[3] *P* values are calculated using the Wald statistic
[4] CI are adjusted for multiple tests for treatment using the Bonferroni procedure; *p*-values are adjusted for multiple tests for treatment using the Holm procedure
[5] Unknown race/ethnicity was excluded from analysis

Supplementary Table 12. Multivariable Probabilistic Index Models for the cumulative Toxicity Index (TI) for the vaginal domain during the acute and subacute time periods. Bolded p values reflect those that are <0.05. A: doxorubicin, BMI: body-mass index, C: cyclophosphamide, CI: confidence interval, NH: non-Hispanic, T: docetaxel.

		Acute (*N*=2068)		Subacute (*N*=1791)	
**Variable**	**Comparison A < B [1]**	**Probability (95% CI) [2]**	***P*-value [3]**	**Probability (95% CI) [2]**	***P*-value [3]**
**Treatment [4]**	AT < ATC	0.483 (0.447, 0.519)	0.642	0.521 (0.484, 0.558)	0.349
	AT < AC -> T	0.502 (0.465, 0.538)	0.919	0.534 (0.497, 0.572)	0.085
	ATC < AC -> T	0.519 (0.483, 0.554)	0.642	0.513 (0.475, 0.551)	0.405
**TI at Baseline**	1-unit increment	0.635 (0.614, 0.655)	**<0.001**	0.626 (0.607, 0.645)	**<0.001**
**Age at surgery (Years)**	1-year increment	0.497 (0.495, 0.498)	**<0.001**	0.498 (0.496, 0.499)	**<0.001**
**BMI (kg/m^2)**	Obesity (BMI >= 30) < Underweight (BMI < 18.5)	0.503 (0.368, 0.637)	0.971	0.433 (0.311, 0.564)	0.318
	Obesity (BMI >= 30) < Normal (18.5 <= BMI < 25)	0.554 (0.524, 0.584)	**<0.001**	0.544 (0.513, 0.576)	**0.006**
	Obesity (BMI >= 30) < Overweight (25 <= BMI < 30)	0.544 (0.513, 0.574)	**0.005**	0.529 (0.497, 0.56)	0.074
**Race/Ethnicity [5]**	NH White < NH Black	0.488 (0.442, 0.535)	0.613	0.44 (0.396, 0.485)	**0.009**
	NH White < Hispanic	0.502 (0.429, 0.575)	0.950	0.469 (0.392, 0.548)	0.446
	NH White < Other	0.433 (0.374, 0.495)	**0.033**	0.508 (0.436, 0.579)	0.833
**Type of surgery**	Lumpectomy < Mastectomy	0.504 (0.48, 0.528)	0.770	0.5 (0.475, 0.525)	0.997
**Endocrine therapy**	No < Yes	N/A		0.525 (0.494, 0.557)	0.119

[1] Comparison A < B denotes the probability that the cumulative TI for B is higher than that for A
[2] Probability of 0.5 indicates no difference between comparisons (i.e., A=B)
[3] *P* values are calculated using the Wald statistic
[4] CI are adjusted for multiple tests for treatment using the Bonferroni procedure; *p*-values are adjusted for multiple tests for treatment using the Holm procedure
[5] Unknown race/ethnicity was excluded from analysis

Supplementary Table 13. Multivariable Probabilistic Index Models for the cumulative Toxicity Index (TI) for vaginal domain individual items during the acute time period Bolded p values reflect those that are <0.05. A: doxorubicin, C: cyclophosphamide, CI: confidence interval, NH: non-Hispanic, T: docetaxel.

		Vaginal Dryness (*N*=2064)		Pain with Intercourse (*N*=1861)	
**Variable**	**Comparison A < B [1]**	**Probability (95% CI) [2]**	***P*-value [3]**	**Probability (95% CI) [2]**	***P*-value [3]**
**Treatment [4]**	AT < ATC	0.491 (0.456, 0.527)	1.000	0.486 (0.453, 0.519)	0.379
	AT < AC -> T	0.496 (0.46, 0.531)	1.000	0.518 (0.485, 0.552)	0.379
	ATC < AC -> T	0.504 (0.469, 0.54)	1.000	0.533 (0.499, 0.566)	0.063
**Symptom at Baseline**	1-unit increment	0.642 (0.618, 0.665)	**<0.001**	0.669 (0.634, 0.702)	**<0.001**
**Age at surgery (Years)**	[57, 80] < [24, 43)	0.555 (0.52, 0.59)	**0.002**	0.583 (0.551, 0.615)	**<0.001**
	[57, 80] < [43, 50)	0.55 (0.518, 0.581)	**0.002**	0.569 (0.54, 0.598)	**<0.001**
	[57, 80] < [50, 57)	0.544 (0.511, 0.577)	**0.008**	0.564 (0.532, 0.594)	**<0.001**
**BMI (kg/m^2)**	Obesity < Underweight	0.526 (0.397, 0.652)	0.692	0.597 (0.474, 0.709)	0.120
	Obesity < Normal	0.554 (0.524, 0.583)	**<0.001**	0.545 (0.517, 0.572)	**0.001**
	Obesity < Overweight	0.538 (0.508, 0.568)	**0.012**	0.543 (0.516, 0.57)	**0.002**
**Race/Ethnicity [5]**	NH White < NH Black	0.499 (0.453, 0.545)	0.955	0.462 (0.424, 0.5)	0.051
	NH White < Hispanic	0.504 (0.431, 0.577)	0.912	0.489 (0.423, 0.555)	0.736
	NH White < Other	0.404 (0.347, 0.465)	**0.002**	0.477 (0.414, 0.541)	0.475
**Type of surgery**	Lumpectomy < Mastectomy	0.504 (0.48, 0.527)	0.768	0.488 (0.466, 0.511)	0.307

[1] Comparison A < B denotes the probability that the cumulative TI for B is higher than that for A

[2] Probability of 0.5 indicates no difference between comparisons (i.e., A=B)

[3] *P* values are calculated using the Wald statistic

[4] CI are adjusted for multiple tests for treatment using the Bonferroni procedure; *p*-values are adjusted for multiple tests for treatment using the Holm procedure

[5] Unknown race/ethnicity was excluded from analysis

Supplementary Table 14. Multivariable Probabilistic Index Models for the cumulative Toxicity Index (TI) for vaginal domain individual items during the subacute time period Bolded p values reflect those that are <0.05. A: doxorubicin, C: cyclophosphamide, CI: confidence interval, NH: non-Hispanic, T: docetaxel.

		Vaginal Dryness (*N*=1775)		Pain with Intercourse (*N*=1563)	
**Variable**	**Comparison A < B [1]**	**Probability (95% CI) [2]**	***P*-value [3]**	**Probability (95% CI) [2]**	***P*-value [3]**
**Treatment [4]**	AT < ATC	0.529 (0.493, 0.565)	0.140	0.521 (0.486, 0.556)	0.231
	AT < AC -> T	0.53 (0.494, 0.566)	0.140	0.545 (0.509, 0.58)	**0.008**
	ATC < AC -> T	0.501 (0.464, 0.538)	0.940	0.524 (0.487, 0.561)	0.231
**Symptom at baseline**	1-unit increment	0.623 (0.601, 0.645)	**<0.001**	0.645 (0.61, 0.678)	**<0.001**
**Age at surgery (Years)**	[57, 80] < [24, 43)	0.508 (0.473, 0.543)	0.644	0.564 (0.529, 0.598)	**<0.001**
	[57, 80] < [43, 50)	0.552 (0.518, 0.585)	**0.002**	0.568 (0.535, 0.6)	**<0.001**
	[57, 80] < [50, 57)	0.538 (0.504, 0.572)	**0.029**	0.535 (0.501, 0.567)	**0.042**
**BMI (kg/m^2)**	Obesity < Underweight	0.462 (0.342, 0.586)	0.550	0.528 (0.418, 0.635)	0.619
	Obesity < Normal	0.545 (0.514, 0.575)	**0.004**	0.548 (0.519, 0.578)	**0.001**
	Obesity < Overweight	0.527 (0.497, 0.558)	0.080	0.527 (0.498, 0.555)	0.072
**Race/Ethnicity [5]**	NH White < NH Black	0.447 (0.404, 0.491)	**0.019**	0.427 (0.389, 0.465)	**<0.001**
	NH White < Hispanic	0.475 (0.405, 0.547)	0.495	0.485 (0.408, 0.563)	0.711
	NH White < Other	0.472 (0.402, 0.544)	0.446	0.516 (0.443, 0.588)	0.674
**Type of surgery**	Lumpectomy < Mastectomy	0.503 (0.478, 0.527)	0.829	0.479 (0.455, 0.503)	0.090
**Endocrine therapy**	No < Yes	0.515 (0.484, 0.545)	0.343	0.543 (0.513, 0.572)	**0.004**

[1] Comparison A < B denotes the probability that the cumulative TI for B is higher than that for A
[2] Probability of 0.5 indicates no difference between comparisons (i.e., A=B)
[3] *P* values are calculated using the Wald statistic
[4] CI are adjusted for multiple tests for treatment using the Bonferroni procedure; *p*-values are adjusted for multiple tests for treatment using the Holm procedure
[5] Unknown race/ethnicity was excluded from analysis
