# Supplementary material for: Toxicity Index, patient-reported outcomes, and persistence of breast cancer chemotherapy-associated side effects in NRG Oncology/NSABP B-30
Source: NPJ Breast Cancer. 2022 Nov 19;8:123. doi: 10.1038/s41523-022-00489-9 (PMC9675799; doi:10.1038/s41523-022-00489-9)
Supplement: Supplementary file 2 — Reporting Summary [file 41523_2022_489_MOESM2_ESM.pdf]

nature portfolio

Corresponding author(s): Henry NL

Last updated by author(s): Aug 8, 2022

Reporting Summary

Nature Portfolio wishes to improve the reproducibility of the work that we publish. This form provides structure for consistency and transparency in reporting. For further information on Nature Portfolio policies, see our Editorial Policies and the Editorial Policy Checklist.

Statistics

For all statistical analyses, confirm that the following items are present in the figure legend, table legend, main text, or Methods section.

n/a	Confirmed	
☐	☒	The exact sample size (*n*) for each experimental group/condition, given as a discrete number and unit of measurement
☒	☐	A statement on whether measurements were taken from distinct samples or whether the same sample was measured repeatedly
☐	☒	The statistical test(s) used AND whether they are one- or two-sided<br>*Only common tests should be described solely by name; describe more complex techniques in the Methods section.*
☐	☒	A description of all covariates tested
☐	☒	A description of any assumptions or corrections, such as tests of normality and adjustment for multiple comparisons
☐	☒	A full description of the statistical parameters including central tendency (e.g. means) or other basic estimates (e.g. regression coefficient) AND variation (e.g. standard deviation) or associated estimates of uncertainty (e.g. confidence intervals)
☒	☐	For null hypothesis testing, the test statistic (e.g. *F*, *t*, *r*) with confidence intervals, effect sizes, degrees of freedom and *P* value noted<br>*Give P values as exact values whenever suitable.*
☒	☐	For Bayesian analysis, information on the choice of priors and Markov chain Monte Carlo settings
☒	☐	For hierarchical and complex designs, identification of the appropriate level for tests and full reporting of outcomes
☒	☐	Estimates of effect sizes (e.g. Cohen's *d*, Pearson's *r*), indicating how they were calculated

Our web collection on statistics for biologists contains articles on many of the points above.

Software and code

Policy information about availability of computer code

Data collection	No software was used
Data analysis	R package version 4.0.5 was used

For manuscripts utilizing custom algorithms or software that are central to the research but not yet described in published literature, software must be made available to editors and reviewers. We strongly encourage code deposition in a community repository (e.g. GitHub). See the Nature Portfolio guidelines for submitting code & software for further information.

Data

Policy information about availability of data

All manuscripts must include a data availability statement. This statement should provide the following information, where applicable:

- Accession codes, unique identifiers, or web links for publicly available datasets
- A description of any restrictions on data availability
- For clinical datasets or third party data, please ensure that the statement adheres to our policy

Individual participant data that underlie the results reported in this article will be made available after de-identification (text, tables, figures, and appendices). The study protocol will also be available. Data will be made available beginning 9 months after publication. Investigators who wish to use the data will have to follow standard NCI NCTC/NCORP Data Archive guidelines for obtaining data, which includes submitting a brief research plan and obtaining a data use agreement between the Data Archive and the investigator's institution (https://nctn-data-archive.nci.nih.gov/about-us).

March 2021

Human research participants

Policy information about studies involving human research participants and Sex and Gender in Research.

Reporting on sex and gender	Only women were eligible to participate in the NRG/NSABP B-30 clinical trial.
Population characteristics	The population characteristics examined in this exploratory analysis were: chemotherapy regimen, age at surgery, body mass index, race/ethnicity, type of breast surgery, and hormonal therapy
Recruitment	Patients were recruited from academic and community-based cancer centers through the National Cancer Institute-supported cooperative groups, including NSABP, the Eastern Cooperative Oncology Group, the Southwest Oncology Group, and the North Central Cancer Treatment Group
Ethics oversight	IRBs at each of the participating institutions provided approval and oversight for the clinical trial

Note that full information on the approval of the study protocol must also be provided in the manuscript.

Field-specific reporting

Please select the one below that is the best fit for your research. If you are not sure, read the appropriate sections before making your selection.

☒ Life sciences ☐ Behavioural & social sciences ☐ Ecological, evolutionary & environmental sciences

For a reference copy of the document with all sections, see nature.com/documents/nr-reporting-summary-flat.pdf

Life sciences study design

All studies must disclose on these points even when the disclosure is negative.

Sample size	A total of 2156 patients were enrolled in the QOL substudy, evenly divided across the three treatment regimens. Of those, 2088 in the acute period and 1802 in the subacute period completed follow-up patient-reported outcomes questionnaires during the time period.
Data exclusions	Patients who did not complete any follow-up questionnaires during the time period were excluded, as shown in Figure 1.
Replication	No replication was performed in this exploratory analysis using data from a previously-conducted large, randomized clinical trial.
Randomization	Patients were randomized 1:1:1 to the three chemotherapy treatment arms in the parent clinical trial. Inclusion in the QOL substudy was limited to the first 2100 consecutively enrolled patients.
Blinding	Blinding was not relevant for this study design, in which probabilistic index modeling was used to examine associations between patient factors and symptoms during the acute and subacute periods.

Reporting for specific materials, systems and methods

We require information from authors about some types of materials, experimental systems and methods used in many studies. Here, indicate whether each material, system or method listed is relevant to your study. If you are not sure if a list item applies to your research, read the appropriate section before selecting a response.

Materials & experimental systems

n/a	Involved in the study	
☒	☐	Antibodies
☒	☐	Eukaryotic cell lines
☒	☐	Palaeontology and archaeology
☒	☐	Animals and other organisms
☐	☒	Clinical data
☒	☐	Dual use research of concern

Methods

n/a	Involved in the study	
☒	☐	ChIP-seq
☒	☐	Flow cytometry
☒	☐	MRI-based neuroimaging

Clinical data

Policy information about clinical studies

All manuscripts should comply with the ICMJE guidelines for publication of clinical research and a completed CONSORT checklist must be included with all submissions.

Clinical trial registration	parent trial clinicaltrials.gov NCT00003782
Study protocol	not relevant for this exploratory analysis
Data collection	data were collected by NSABP for the parent trial
Outcomes	not applicable for this exploratory analysis
